# Neurocysticercosis in a Japanese woman with lung cancer who repeatedly visited endemic countries

**DOI:** 10.1186/s12879-021-06778-1

**Published:** 2021-10-18

**Authors:** Tomoya Kinouchi, Yasuyuki Morishima, Shinichi Uyama, Tadashi Miyamoto, Hidehisa Horiguchi, Naomi Fujimoto, Hiromi Ueta

**Affiliations:** 1 Department of Neurosurgery, Tokushima Municipal Hospital, 2-34, Kitajyosanjima-cho, Tokushima, 770-0812 Japan; 2 Department of Parasitology, National Institute of Infectious Diseases, Tokyo, Japan; 3 Department of Laboratory Medicine and Pathology, Tokushima Municipal Hospital, Tokushima, Japan; 4 Department of Neurosurgery, Tokushima Kensei Hospital, Tokushima, Japan

**Keywords:** Neurocysticercosis, Metastatic brain tumors, Albendazole, Developing countries

## Abstract

**Background:**

*Taenia solium*, present in most developing countries, infects many individuals and may result in their death. Neurocysticercosis (NCC) develops after invasion of the brain by parasitic larvae. It is the most common parasitic disease of the human central nervous system. On imaging scans it can be similar to brain tumors. We report a patient with a metastatic brain tumor and NCC. The co-presence of NCC was diagnosed based on specific neuroimaging- and epidemiologic findings.

**Case presentation:**

A 36-year-old non-smoking Japanese woman with a history of non-small-cell lung cancer had undergone resection of the lower lobe followed by cytotoxic chemotherapy 2 years before apparently suffering recurrence. A positron emission computed tomography (PET) scan incidentally revealed multiple intracranial cold spots exhibiting differences in their shape and size. On brain magnetic resonance imaging (MRI) scans we observed many different patterns of peripheral edema and gadolinium-enhancing effects. As she had often visited Latin America and Southeast Asia and had eaten raw pork and Kimchi, we suspected that the brain lesions were due to NCC rather than metastatic brain tumors. However, serum immunoblotting assay and DNA analysis were negative for *T. solium*. Rather than performing resection, we administered albendazole (ABZ) and dexamethasone because her earlier cytotoxic chemotherapy had elicited severe pancytopenia. Except for a single large lesion in the left frontal lobe, this treatment resulted in a significant reduction in the size of these lesions and a decrease in perilesional edema. She underwent resection of the residual lesion 10 months later. Histology revealed it to be a metastatic tumor. Polymerase chain reaction (PCR) assay for NCC was negative. In the course of 11-months follow-up there has been no recurrence.

**Conclusion:**

This is the first presentation of NCC in a Japanese woman with a metastatic brain tumor. NCC was incidentally discovered on PET scans and, based on her travel history and epidemiological findings; it was diagnosed and successfully treated with ABZ. NCC is endemic in most developing countries and as visits to such countries have increased, NCC must be ruled out in patients with multiple cystic nodular brain lesions.

## Background

Encysted larvae of the tapeworm *Taenia solium* are responsible for the development of neurocysticercosis (NCC), a parasitic infection of the central nervous system (CNS). NCC, a major cause of acquired epilepsy and neurological morbidity, is endemic in most developing countries where healthy individuals may become infected by drinking water contaminated by feces harboring tapeworms or by eating contaminated vegetables or raw pork. As visits to- and prolonged sojourns in developing countries have become popular, the rate of *T. solium* infection of travelers can be expected to rise. On neuroimaging studies, NCC is characterized by multiple intracranial cysts in different stages of development. When patients with pulmonary carcinoma report having traveled to endemic areas, it is difficult to work up the etiology of their multiple intracranial cystic lesions.

## Case presentation

Two years before consulting us, a 36-year-old Japanese woman without a relevant medical history developed systemic joint pain. A chest radiogram acquired at a local hospital revealed a tumor in the left lower lobe; its diameter was 3-cm. She was referred to Department of Respiratory Medicine at our hospital and underwent bronchoscopy; cytology of the tumor resulted in a diagnosis of adenocarcinoma. Gadolinium-enhanced brain MRI scans and bone scintigraphy revealed no metastases. The tumor was clinically classified as T1N1M0, stageIIa. The lower lobe was resected and cytotoxic chemotherapy was administered. Two years later she presented with recurrence and received carboplatin, pemetrexed, and pembrolizumab (immune checkpoint inhibitor) therapy.

**Fig. 1:**
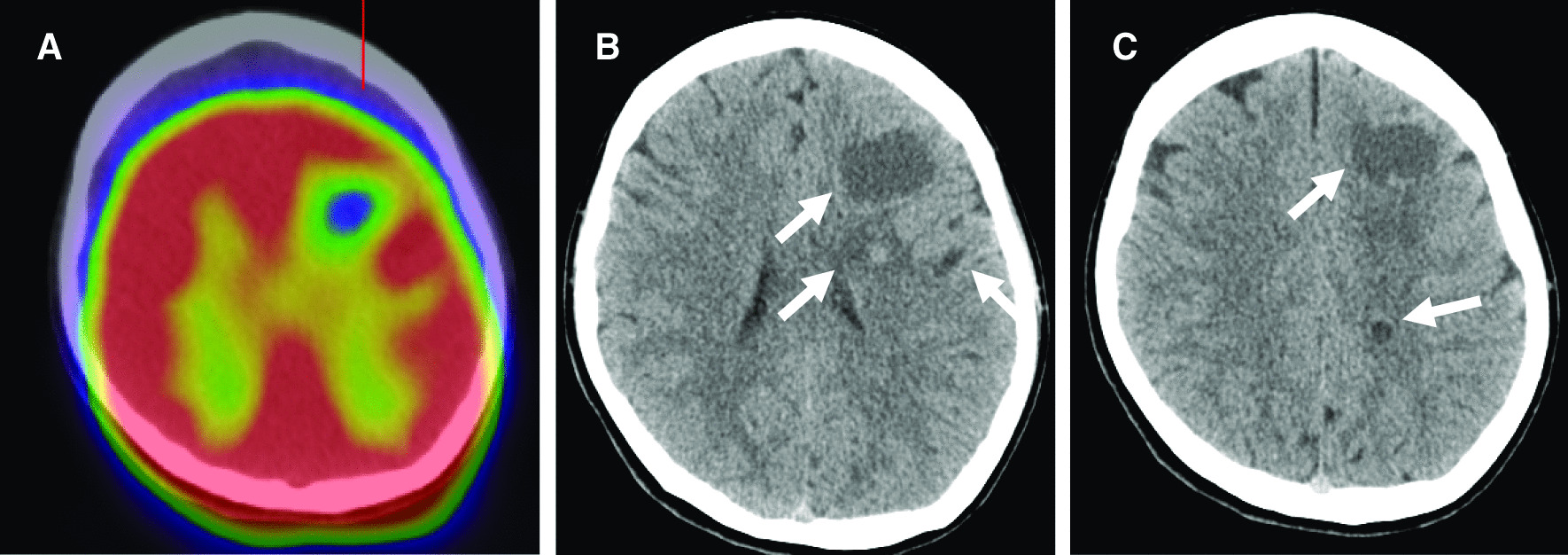
**A** PET revealed intracranial cold spots. **B**, **C** Brain CT scans showing cystic lesions without calcification in the parenchyma of the left hemisphere (arrows)

**Fig. 2:**
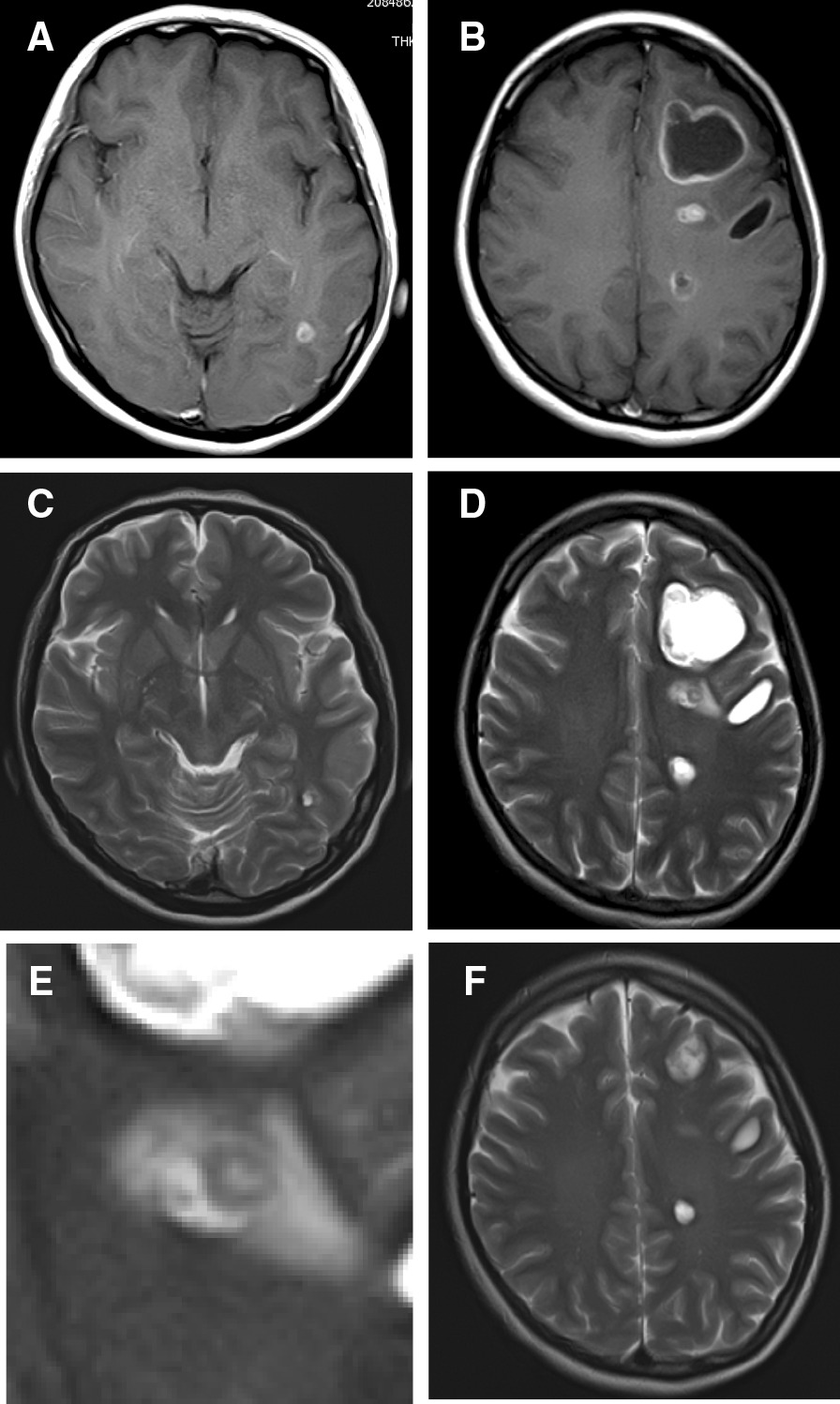
Brain MRI findings. **A**, **B** Gadolinium-enhanced brain MRI revealed strong ring enhancement of cystic lesions. **C**, **D** T2-weighted image showing some perilesional focal edema. **E** T2-weighted image revealing a scolex inside the cyst located outside the left lateral ventricle. **F** A brain MRI scan acquired 7 months after the start of ABZ treatment demonstrated multiple small well-defined cystic and nodular structures of different sizes without perilesional edema or calcification

A PET scan incidentally revealed intracranial cold spots (Fig. 1A) and a CT scan disclosed multiple cystic lesions without calcification in the parenchyma of the left hemisphere; they measured 10–35 mm in diameter (Fig. 1B, C). On MRI scans most of the cysts were ring-enhancing (Fig. 2A, B) and some were surrounded by perilesional edema (Fig. 2C, D).

An eccentric nodule observed on a T2-weighted image of cysts outside the left lateral ventricle appeared to be a scolex (Fig. 2D, E). On MRI scans, the size of the lesions had increased very slowly in the course of 7 months (Fig. 2F). As single-photon emission CT (SPECT) denied the accumulation of thallium-201, a definitive diagnosis of malignant tumor could not be made.

**Fig. 3:**
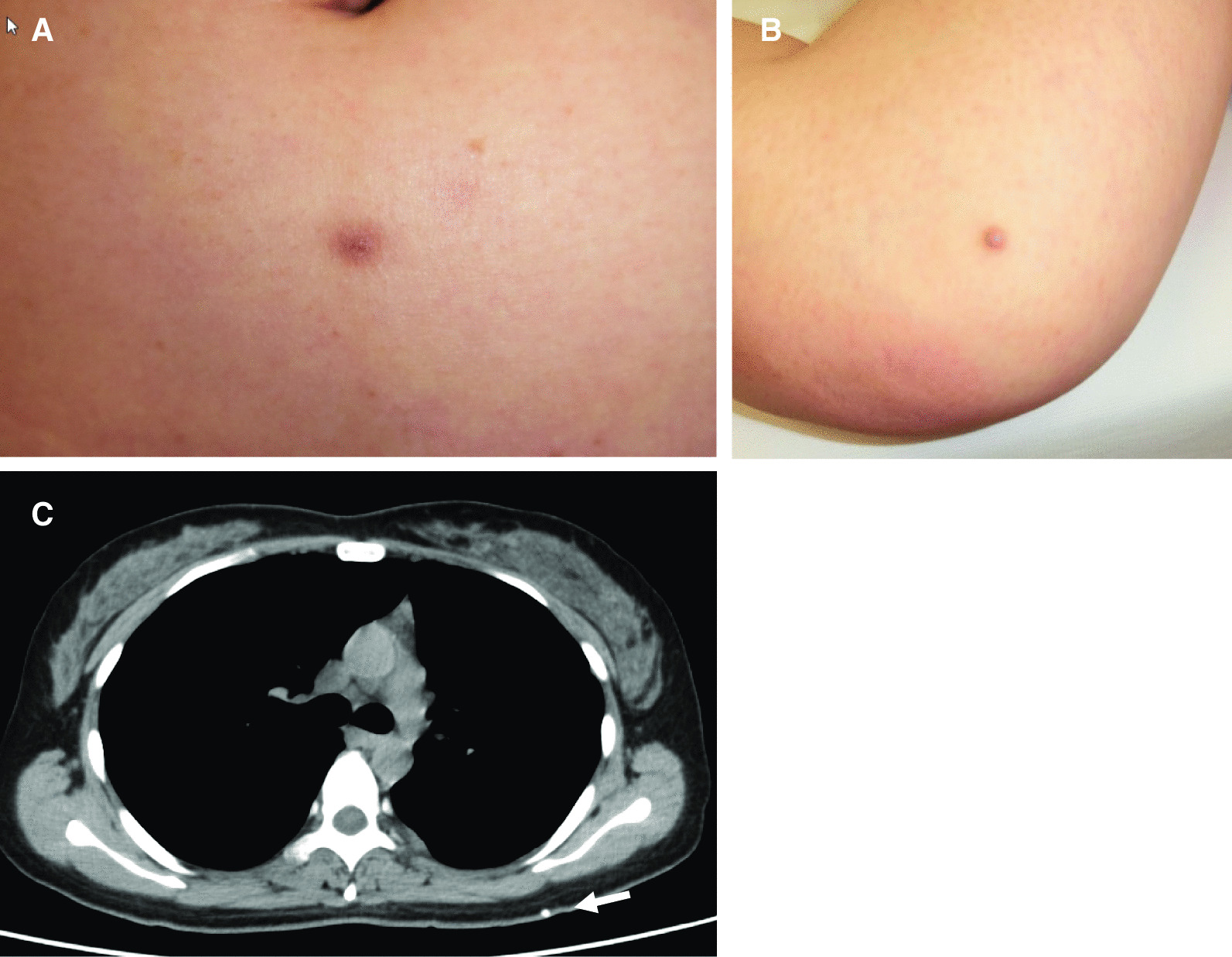
**A**, **B** On physical examination, more than 10 subcutaneous lesions were palpable on the chest, back, lower abdomen, and right knee. **C** Chest CT scan showing a calcified lesion high on the back (arrow)

At the time of admission to our Neurosurgery department, her consciousness was clear. She had no significant family- or employment history. She did not smoke or drink alcohol. Her blood pressure was 125/72 mmHg, her pulse rate was 68/min, and her temperature was 36.8 °C. Neurological examination revealed slight motor aphasia. Physical examination showed that more than 10 subcutaneous lesions were palpable on her chest, back, lower abdomen (Fig. 3A) and her right knee (Fig. 3B). Chest CT revealed that one of the lesions on her back was calcified (Fig. 3C).

In a patient interview she reported that between 2009 and 2018 she had visited Canada, the USA, Bolivia, Peru, Chile, Cambodia, the Philippines, Indonesia, New Zealand, and Australia and that each sojourn lasted from 3 weeks to several months. In 2012 and 2013 she had often eaten whole roasted young pig in the Philippines and Korean Kimchi during her 2013–2014 visit to Canada. Based on this information we suspected that she may have become infected with cysticercosis in the endemic areas she visited.

We started treatment with the anti-epileptic drug levetiracetam (1000 mg/day) for seizure prophylaxis and asked for a cysticercosis analysis. Both DNA analysis from biopsy specimens from her right knee and lower abdomen and serum immunoblotting assay performed at the National Institute of Infectious Diseases (NIID) were negative for cysticercosis. We rejected cystic lesion biopsy because molecularly-targeted therapy with crizotinib had elicited strong pancytopenia. Instead, we administered ABZ (400 mg twice per day at 15 mg/kg/day), and decadron (4 mg twice per day at 0.1 mg/kg/day). However, we interrupted this regimen 5 days later because she developed fever, headache, a generalized rash, and swelling of the oral mucosa; we thought them to be reactions to treatment with ABZ.

**Fig. 4:**
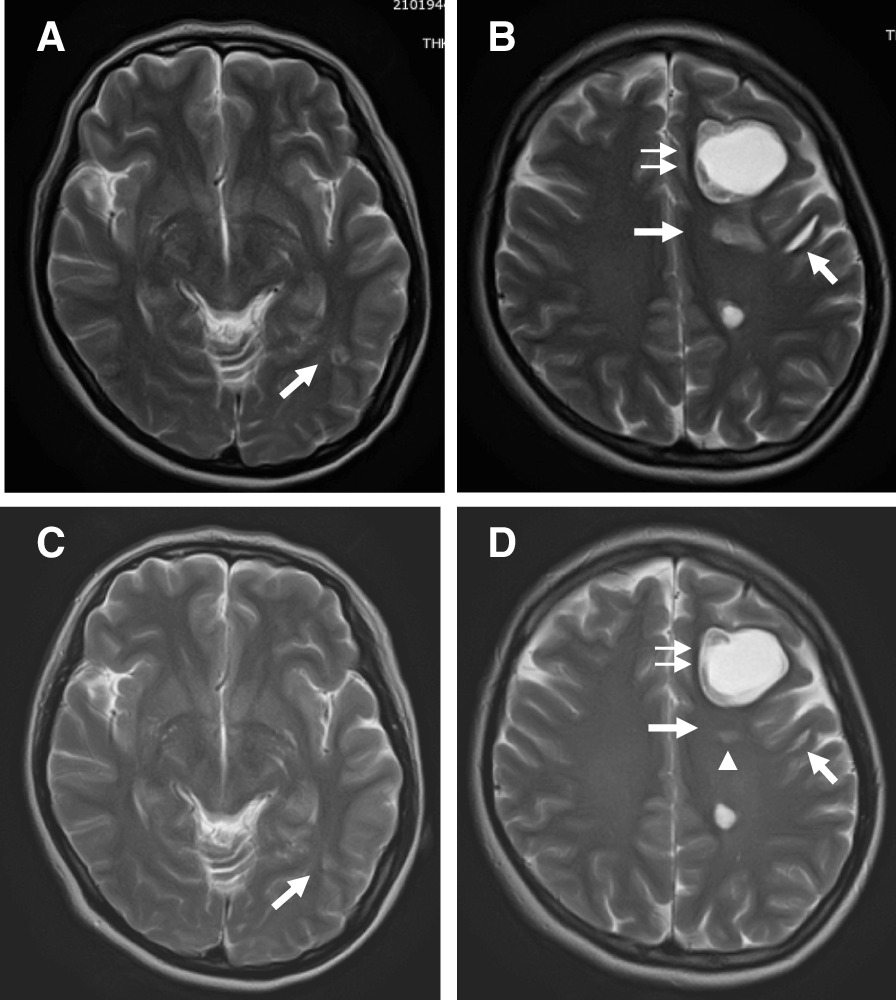
T2-weighted brain MRI scans obtained 7- (**A**, select all **B**) and 30-days (**C**, **D**) after the start of cysticidal treatment. Except for the largest, the size of almost all lesions in the frontal lobe was decreased as was perilesional edema (arrows). There is no change in the left frontal lesion (double arrows). The scolex inside the cyst located outside the left lateral ventricle has disappeared (arrowhead)

Follow-up MRI studies performed 1 week after the start of treatment showed that the size of many of the cystic brain lesions had shrunk and that perilesional edema had decreased (Fig. 4A, B). With the exception of the large left frontal mass, most lesions had resolved on day 30 and no tapeworm scolex was detected (Fig. 4C, D). We continued treatment with crizotinib.

Follow-up 10 months later revealed no new lesions or seizures. Her lung adenocarcinoma was controlled without crizotinib. However, the left frontal cystic lesion had not regressed. During the 10-month interval she frequently suffered left frontal headaches and motor aphasia. Considering them to be symptoms of intracranial hypertension and a mass effect induced by the lesion located at a depth of 10 mm subcortically, we resected almost all of the lesion. It was thin-walled, well delineated from surrounding brain tissue, and contained a yellow, pus-like fluid. No cysticericus was found. Histological examination revealed metastasis from the lung adenocarcinoma; PCR assay for NCC returned negative results. The cell count in the pus-like fluid was 24,000, bacteriology rendered no findings. A gamma-knife procedure was applied around the operative scar in the left frontal lobe. Crizotinib administration was continued and she reported no headaches, no aphasia and her lung adenocarcinoma did not recur. A brain MRI scan performed 11-months after her admission to our hospital confirmed the absence of active and/or degenerative lesions.

## Discussion

This is the first documentation of co-existing NCC in a Japanese woman with a metastatic brain tumor. NCC was incidentally diagnosed based on neuroimaging scans and upon epidemiological investigations. Treatment with ABZ was successful. NCC is the most common helminthic infection of the CNS; in endemic countries (e.g. Latin America, sub-Saharan Africa, and parts of Asia other than Japan) it often leads to seizures [1, 2].

NCC is the result of infection with the larval stage of the tapeworm *T. solium*. The infection occurs when eggs excreted in the feces of individuals carrying the parasite are ingested through contaminated water, and via the ingestion of raw and/or undercooked food. It can also come directly from a carrier via the oral‐fecal route [1–4]. The ingestion of contaminated food harboring active *T. solium* cysticerci can elicit *T. solium* taeniasis, a condition in which the adult tapeworm is found in the human intestines [5]. Eggs of the adult tapeworm are subsequently shed in stool, completing the cycle.

NCC can mimic metastatic brain tumors, toxoplasmosis, and tuberculosis on imaging scans. In tropical countries it is more often encountered than brain tumors. Others [6, 7] reported that international travelers from non-endemic countries developed NCC after visiting endemic areas. Such cases were diagnosed based on pathological or serological findings. Most infected individuals developed symptoms 2 or more years after returning home [6] and cysticerci have been reported to survive silently for more than 10 years [8].

In our patient we initially suspected the lesion to be a metastatic brain tumor because she had received chemotherapy for non-small-cell lung cancer. However, three observations led us to suspect something other than a metastatic brain tumor: The size of her multiple lesions increased very slowly compared to a malignant tumor, unlike metastatic brain tumors that usually manifest as solid masses, hers contained a cystic component, and the shape of the masses and the paralesional edema around them exhibited many different patterns.

We think that our radiographical studies revealed different evolutionary stages of NCC. Neuroimaging studies are essential for the diagnosis of NCC and MRI- have been shown to be superior to CT studies with respect to the detection and characterization of NCC [9]. According to the NCC classification on neuroimaging studies, there are four main developmental stages [9–13]. In the vesicular stage of viable larvae there is no surrounding vasogenic edema; only very faint enhancing lesions are observed. In the colloidal vesicular stage, the lesions are hyper-intense vis-à-vis the CSF on CT- and T1-weighted images and surrounding edema is present. The nodular wall then thickens, enhances brightly, and the scolex is observable. In the granular nodular stage enhancement persists but is less marked and edema surrounding the cyst decreases. In the nodular calcified stage, i.e. the end-stage, the nodule is quiescent and calcified and there is signal drop-out on T2-weighted images. We hypothesize that in our patient, neuroimaging revealed transitional periods from the vesicular- to the colloidal vesicular stage.

Del Brutto et al. [14, 15] proposed criteria for the diagnosis of NNC. In diagnosing our patient we considered findings on imaging scans, the observation of cystic lesions with or without a discernible scolex, lesion enhancement, and clinical/exposure information although the presence of the eccentric nodule in the cyst outside the left lateral ventricle and the observation of a scolex on T2-weighted images alone are sufficient for a definitive NCC diagnosis.

Although neither serum immunoblotting assay, DNA analysis, nor NIID results were positive for *T. solium*, we administered antiparasitic drugs. ABZ and praziquantel (PZQ) have been reported to be effective in patients with NCC [16–18]. ABZ enters the CSF and its concentration is not affected when steroids are also administered [19, 20]. Based on trials in adults with viable cysts, Abba et al. [21] suggested that ABZ reduced the number of lesions. However, such drug treatments may be associated with severe adverse reactions due to the release of cyst-destroying antigens and may elicit local tissue swelling and a generalized reaction [22].

In our patient, MRI studies performed 7 days after the start of ABZ therapy revealed a significant reduction in the size of all brain lesions except the largest lesion in the left frontal lobe. Over time, almost all perilesional edema completely resolved; the scolex also disappeared. The resolution on neuroimaging scans of cystic lesions after cysticidal drug therapy is a diagnostic criterion for NCC [14, 15]. For the definitive diagnosis of our patient we used neuroimaging- and clinical/exposure data.

Our patient’s multiple cystic brain lesions consisted of a mixture of metastases from a primary non-small-cell lung cancer and NCC. We think that her transient fever, headache, systemic rash, and swelling of the oral mucosa seen on day 5 of ABZ treatment reflected an inflammatory reaction.

## Conclusion

This is the first presentation of NCC in a Japanese woman with a metastatic brain tumor. NCC was incidentally discovered on PET scans and, based on her travel history and epidemiological findings; it was diagnosed and successfully treated with ABZ. As the number of international travelers has increased, diagnosticians must consider the possibility of NCC when individuals, having spent time in areas where *T. solium* is endemic, present with multiple cystic nodular brain lesions.

## Data Availability

Not applicable.

## Abbreviations

NCC: Neurocysticercosis
PET: Positron emission computed tomography
CNS: Central nervous system
MRI: Magnetic resonance imaging
CT: Computed tomography
ABZ: Albendazole
PCR: Polymerase chain reaction
NIID: National Institute of Infectious Diseases
